# Evaluating the Role of Social Capital in the Sustainable Use of Common-Pool Resource Use: Evidence from Participatory Forest Management in Ethiopia

**DOI:** 10.1007/s00267-026-02447-8

**Published:** 2026-04-16

**Authors:** Yeshimebet Ayele Tegenie

**Affiliations:** 1 Development Economics Chair Group, Wageningen University and Research, Wageningen, Netherlands; 2 Forest Ecology and Forest Management Chair Group, Wageningen University and Research, Wageningen, Netherlands; 3 College of Agriculture and Environmental Sciences, Arsi University, Asella, Ethiopia

**Keywords:** Social capital, Participatory forest management, Resource extraction, sustainability, Ethiopia

## Abstract

The concept of social capital has gained considerable attention as an important tool for fostering collective action in the management of common pool resources (CPRs), such as forests. However, social capital is inherently context-dependent, and in contexts where Indigenous communities hold insecure property rights over CPRs, research on how social capital influences resource use decisions remains limited. This study investigates how the cognitive and bonding dimensions of social capital within communities—measured through household surveys—affect forest resource use decisions. The study first predicted theoretically optimum levels of forest resource extraction under both long- and short-term tenure arrangements. It uses the values predicted under the long-term property rights as optimal sustainable extraction. A framed field experiment was conducted in Ethiopia, where participants from communities played resource extraction games in groups under short-term property rights. A group of individuals voted unanimously to establish a non-binding extraction target before making individual extraction decisions, which were either publicly disclosed or kept private. The findings show that the cognitive dimension tends to increase the temptation to set group extraction targets higher than the optimum sustainable level, while the bonding dimension leads to lower group extraction targets as the resource becomes scarcer. Additionally, the cognitive dimension prompts individuals to focus on short-term gains (i.e., leading to more extraction in early rounds). Its influence remains consistent regardless of whether individual extraction decisions are publicly disclosed. In contrast, bonding dimension has no effect. The study concludes that social capital’s influence is more context-dependent than anticipated.

## Introduction

In the context of collective action for sustainable management of natural resources, it is increasingly recognized that social capital plays a pivotal role (Ballet et al., 2007; Krishna & Uphoff, 1999; Pretty, 2003; Pretty & Smith, 2004). Social capital comprises features of social organization such as generalized trust, and access to and membership in various types of networks (Putnam, 1993a). The premise supporting the importance of social capital was built on the idea that social capital (e.g., social networks) enables better communication and improves access to information (Lauber et al., 2008; Putnam, 1993a). Communication elevates trust and strengthens social cohesion, and vice versa (Ben-Ner & Putterman, 2009; Valley et al., 1998). Enhanced trust reduces opportunistic behavior and transaction costs of cooperation, provides insurance against the risk and uncertainties of cooperative dilemmas, and, thus, allows dilemmas of collective action to be solved (Edwards et al., 2009; Fukuyama, 1995; Lin, 2002; Pretty, 2003; Woolcock & Narayan, 2000).

Scholars have increasingly been using the idea of social capital to describe economic behaviors in natural resource management. For example, based on data from artefactual experiments and surveys, a study in Burundi by Beekman and Bulte (2012) shows a positive link between social capital (e.g., trust) and investments in soil erosion control. Similarly, a study conducted in India using artefactual experiments and household surveys found a correlation between trust and the maintenance of soil and water conservation (Bouma et al., 2008). Knuffman (2011) uses data from framed-field experiments and household surveys to establish a causal relationship between trust and groundwater extraction in India, providing evidence that social capital has a direct impact on individuals’ behavior. A case study in Sri Lanka also demonstrates a causal relationship between social capital and irrigation management (Aida, 2019). Moreover, a study by Baylis et al. (2018) shows a causal impact of bridging and bonding social capital on fuel wood collection in China.

Social capital is context-dependent, as it is influenced by culture, location, social structure, and government structures (Ballet et al., 2007; Baycan & Oner, 2022; Krishna & Shrader, 1999; Li et al., 2019). While its context-specific nature has been widely recognized, no research has examined its role in the sustainable use of common-pool resources managed by community-based associations under conditions of insecure property rights and information transparency. Using Ethiopia as a case study, this study therefore aims to explore the relationship of social capital with collective and individual decision-making regarding sustainable resource extraction in situations where users hold short-term property rights, and their decisions are either publicly disclosed or kept private.

I acknowledge that focusing on a single national context presents both opportunities and challenges. It enables an in-depth, context-sensitive examination of locally embedded institutional and cultural dynamics that broader comparative studies may overlook. At the same time, findings from a single country — and specific regions within it — may have limited generalizability and may not capture all intra-country variation. Despite these limitations, the study aims to provide contextually grounded insights that contribute to broader theoretical discussions.

This focus is particularly important in Ethiopia, where, despite government efforts to promote community-based forest resource management, dryland forests continue to face pressure from unsustainable use (Bongers et al., 2019; Lemenih et al., 2014). Insecure tenure arrangements were noted as one of the causes of sustained overutilization of common property resources, especially frankincense-bearing ones (Eshete et al., 2021; Gebru et al., 2014; Hagazi, 2012). The fact that different users with divergent interests share the same natural resource explains why common resource management leads to free-rider behaviors that impede the sustainable use of resources (Ostrom et al., 1994). This issue is further aggravated by short-term tenure arrangements (Tegenie et al., 2024). However, theoretically, it is claimed that local communities as organizations are capable of solving this problem as long as they institute informal institutions that reduce opportunistic behaviors and guarantee the enforcement of collective actions (Ballet et al., 2007; Pretty, 2003; Pretty & Smith, 2004). In Ethiopia, in general, and in the drylands in particular, rural communities rely on informal institutions to enforce rules and cope with livelihood challenges (Degefa, 2010; Jang et al., 2016; Woldeamanuel, 2011). In addition, the livelihood resilience of Ethiopian dryland communities depends heavily on the income from dryland forest resources, particularly woodlands that produce frankincense (Lawry et al., 2015; Lemenih & Kassa, 2010; Worku et al., 2014), which are heading to extinction (Bongers et al., 2019). Therefore, it is timely to look into whether and how social capital, as an informal institution, contributes to the sustainable use of shared forest resources managed under a short-term property regime.

The study is based on a framed field experiment conducted in the Benishangul-Gumuz Regional State of Ethiopia. In collaboration with an international organization (Farm Africa), the government of Benishangul-Gumuz introduced a participatory forest management (PFM) program (Lemenih et al., 2015).^1^ This program led the formation of local community-based associations or cooperatives. These cooperatives lease *Boswellia papyrifera* forests, the source of frankincense, from governmental agencies based on concessions that are typically for one year. 2 Members of associations take collective responsibility for the management of the resource. They also have the right to extract non-timber forest products, including frankincense. The field experiment was conducted among these association members, all of whom extract frankincense for commercial purposes. In the framed field experiment, groups of participants set extraction targets collectively through unanimous voting and make individual extraction decisions. The data for this chapter concerns a sub-sample of participants that were randomly assigned to the treatment conditions with short-term property rights regime, either with or without disclosing their choices.^3^ Finally, the study measures the cognitive and structural bonding dimensions of social capital using a household survey.

This study fits within the broader economic research theme that investigates the role of social capital as a central guiding force for the sustainable use of natural resources, or CPR, and, consequently, economic development (Fukuyama, 2002; Knack & Keefer, 1997; Woolcock, 1998; Woolcock & Narayan, 2000). The findings of the study show that the cognitive dimension of social capital fosters cooperation, but this does not translate to sustainability; it increases the temptation to set extraction targets above the optimum level and influences individuals to focus on short-term gains. While structural bonding social capital facilitates cooperation in collective decision-making, which can be translated into sustainability, it does not influence individual decisions. Moreover, the findings reveal that information disclosure does not affect the influence of either cognitive or structural bonding social capital.

This paper has two contributions. First, it provides a novel perspective on how social capital functions in less secure tenure and information-transparent environments, thereby offering practical insights for policymakers and practitioners. Second, it considers the role of the cognitive dimensions of social capital, while most existing literature focuses on bonding and bridging.

## The Concept of Social Capital

The concept of social capital has evolved through the contributions of several scholars, with Bourdieu (1986) and Coleman (1988) providing the first systematic definitions. In Bourdieu’s work, social capital is defined as “the aggregate of the actual or potential resources that are linked to the possession of a durable network of more or less institutionalized relationships of mutual acquaintance and recognition — in other words, to membership in a group” (Bourdieu, 1986). Bourdieu’s work highlights the importance of social ties in the acquisition of opportunities and benefits. In Coleman’s work, however, social capital refers to elements of the social structure that enable specific actions by individuals within that structure (Coleman, 1988, 1994). Coleman identifies trust and reciprocity, information channels and the flow of information, and norms enforced by sanctions as three forms of social capital. Putnam (1993a) popularized the term by describing social capital as “features of social organization such as trust, norms, and networks that can improve the efficiency of society by facilitating coordination and cooperation for mutual benefits.” For Putnam, social networks hold value, and social connections that influence the outcomes of both individuals and groups.

Building on mainstream research on social capital (e.g., Bourdieu (1986), Coleman (1988), Putnam (1993a)), Uphoff (2000) categorized social capital into two broader dimensions: cognitive and structural dimensions.^4^ The structural dimension is linked to various forms of social organizational-specific rules, precedents, and procedures, and a diverse range of networks that foster cooperation for commonly beneficial collective actions (Colletta & Cullen, 2002; Gooderham, 2007; Nahapiet & Ghoshal, 1998). Structural social capital is further classified into three categories: bonding, bridging, and linking. Bonding social capital refers to the ties between individuals or groups within a community (Woolcock & Sweetser, 2002), while bridging structural social capital refers to ties between groups or communities and tends to connect people across diverse social divisions (Baker, 1990; Field, 2008; Woolcock, 2001). Both bonding and bridging reflect horizontal connections for people of similar status and power (Aldrich, 2011; Putnam, 1993a). Finally, linking structural social capital relates to vertical connections with formal institutions to influence their policies or access valuable resources (Pretty & Ward, 2001; Woolcock, 1998, 2001).

Cognitive social capital stems from a mental process and is strengthened by culture, ideology, values, beliefs, and norms that promote cooperation in collective actions (Uphoff, 2000). Values include trust, reciprocity, and norms that are shared among members of a community and create the conditions under which communities can work together for a common good (Krishna & Shrader, 1999). Trust is having confidence that the group will function as intended (Pretty, 2003; Pretty & Ward, 2001). Reciprocity involves both internalized personal moral norms and a pattern of social exchange (Ostrom & Ahn, 2009a; Pretty, 2003; Pretty & Ward, 2001). It fosters long-term obligations between individuals, which are crucial for achieving positive environmental outcomes (Pretty & Smith, 2004). Social norms refer to a set of beliefs about acceptable and unacceptable behavior, and they are usually called the rules of the game (Ostrom, 1990; Taylor, 1982). This gives rise to some kind of social control over what forms of behavior are socially accepted and valued (Luo et al., 2018).

Additionally, social capital is thought to exist at various levels within society’s hierarchy. Consequently, it can be measured and analyzed from a social perspective at the individual and collective levels, as well as from a geographic perspective at the micro and macro levels (Bourdieu, 1986; Bowles & Gintis, 2002; Brehm & Rahn, 1997; Chen, 2005; Coleman, 1994; Glaeser et al., 2002; Glanville et al., 2016; Ostrom & Ahn, 2009b; Turner, 2000). Furthermore, empirical measures of social capital involve various indicators. Putnam, amongst others, used the density of membership in voluntary associations of all kinds as a proxy for social capital (Paxton, 2002; Putnam, 1993b; Putnam, 1995). This employs a limited set of data and points to structural social capital. Krishna et al. (1999) argue that measures of social capital will depend on the country and culture one is studying. In their study conducted in India, they used a range of questions on trust, norms, reciprocity, dealing with disasters, and membership in labor-saving groups as a measure of social capital. Other studies also create a score by combining a small set of different indicators obtained through survey sample questions (Diaz et al., 2002; Sharp & Smith, 2003).

## Conceptual Framework

Studies of collective action in community-based natural resource management have widely employed the social capital framework (Anderson et al., 2002; Baldassarri, 2015; Barnes-Mauthe et al., 2013; Baylis et al., 2018; Bodin & Crona, 2008). Similar to earlier studies, this study uses the social capital framework to conceptualize the relationship between social capital and shared forest use. To do so, this section begins by presenting a model developed to evaluate institutional arrangements aimed at achieving sustainable production of forest resources (for more detail see Tegenie et al. (2024)). It then presents how cognitive and bonding dimensions of social capital influence group or individual decisions in collective action.

### Extraction Model

The model is based on the assumption that individual extraction strategies exist for communal forest resources. Individuals have use rights over forest resources, and they are involved in gum and resin production, specifically frankincense, for commercial purposes. The model is described by (1) a harvest function that describes the extraction level (the link between the level of wounding and harvest), which also determines the health of the resource (and future yields), (ii) a cost of effort function, and (iii) a payoff function that links harvest, prices, and costs.

The model describes that at each period $t$, individuals enter the forest to cut the bark of frankincense-bearing trees in a circular form along the stem to release and collect the frankincense. It assumes that individuals choose the number of wounds to apply at period $t$ based on the health of the tree. The model represents the health of a tree and the number of wounds applied at period $t$ by ${V}_{t}$ and ${q}_{t}$, respectively. The harvest from a tree at period $t$, ɦ_*t*_ ≥ 0, is given as:1where t = 1, 2, 3, …, n represents harvesting periods, and ƶ is a constant representing the production coefficient of wounding the frankincense-bearing tree.

The model considers a tree’s capacity to bear resins as a measure of its health, and it is then given as:2where ɑ is a constant that measures the health of a tree without wounding (the initial health of the tree), ɓ is the marginal effect of wounding a tree on its health, and *Q*_*t*_ is the cumulative wounds applied during the life time of the tree until (including) time *t* and given as:

3 $${Q}_{t}=\mathop{\sum }\limits_{i=1}^{t}{q}_{i}.$$

In practice, there are a fixed number of wounds that are repeatedly opened on the same tree. Moreover, frankincense extraction follows a cyclic process. Within the same harvesting season (year), initial wounds opened at the start of period *t* are reopened (refreshed) and slightly widened by removing additional bark. In the following harvesting season, instead of revisiting the old wounds, new ones are opened. Hence, increasing the number of wounds decreases the marginal returns of that spot already during that year, as it affects both the current and future tree health through this repeated opening (widening) of the same wound. In the model, this is described by introducing the negative health effects of an extra cut. Combining (2) and (3), the harvest function is then rewritten as:4

In line with empirical evidence (Cherenet et al., 2020; Eshete et al., 2012), the harvest function is exhibiting diminishing harvest levels with the number of wounds applied to a tree. The cost of extraction at time *t* (${C}_{t}$) is explained in terms of a unit effort spent in wounding trees, $c{q}_{t}$, where $c$ represents the marginal effort per unit wound. A unit effort means the time spent on the extractive activity, which is represented in days in the model. The marginal effort per unit wound is set to 0.5 and remains constant across periods. 5 The cost function is then given as:

5 $${C}_{t}=0.5{q}_{t}.$$

The model describes that individuals harvest frankincense for profit. Since both harvest and costs depend on the number of wounds, individuals choose wound levels that maximize their payoffs. The model was developed based on the assumption that individuals are risk-neutral and have complete information about tree health at each period. The harvested frankincense is also sold in a competitive market at a price of *P* > 0. An individual’s payoff function at period $t$ is modeled as:6

The payoff function takes a quadratic shape, reflecting decreasing marginal returns in CPR (Ostrom et al., 1994). The optimal wounding levels that maximize an individual’s payoff were first derived under both long- and short-term property rights regimes, and then the predicted optimal values are used as a benchmark to describe the collective choice treatment and the influences of social capital on a group and individuals’ choices.

In this paper, a long-term property rights regime refers to a situation where the adverse impact of cutting on the tree over its lifespan is fully internalized (i.e., without additional incentives) by the forest users in their decision-making. Let a group of five homogenous individuals manage five trees for harvesting frankincense, where each individual has property rights over one tree and uses the same tree over its lifespan. In other words, there is no shuffling of trees between group members in between cutting periods, hereafter referred to as the no shuffling scenario. In the model, the time horizon of extraction is limited to three periods, after which the tree is exhausted. Individuals anonymously choose the number of wounds they apply in each of the three periods. Using the first-order condition, the model predicts that the optimal number of wounds applied to trees under the no shuffling scenario remains constant over time and is given as:7

The extraction levels are greater than zero if Pƶα > ½. That means the feasibility of profitable extraction increases with the price of frankincense, the production coefficient, and the initial health of the tree.

Conversely, a short-term property rights regime refers to a setting where exclusive rights are limited to just one period, and the trees are randomly reallocated or shuffled among the group members for the next period, hereafter referred to as the shuffling scenario. In this scenario, the setup of the CPR extraction model remains similar with no shuffling in all other aspects. Harvesters collect frankincense in the absence of monitoring and sanction mechanisms to deter the overuse of CPR. In contrast to the no-shuffling treatment, individuals independently maximize a one-time payoff from a tree, while the costs due to damage are shared by all group members. The optimal number of wounds is given as:8

Note that ${q}_{1}$ > ${q}_{2}$ > ${q}_{3}$, if Pza > ½.

In general, the model predictions show that the overall cuts are lower under long-term property rights. The focus on first-round profits (when the tree is still healthy) under the shuffling regime comes at a cost, as it negatively affects harvests and the sustainability of the resource in the long term.

### Social Capital and Collective Choice

Within the shuffling scenario, there is a social dilemma where extraction is based only on individual rationality, and negative externalities within a group are completely ignored (Dawes, 1980). Group members directly benefit from investing effort in extracting the communal resource. However, as aggregate extraction increases, individuals’ payoffs per period rapidly decrease compared to the no-shuffling scenario, reflecting negative externalities such as deterioration of the health of trees or biodiversity loss in the long run.

In real-life situations, communities develop exploitation plans, such as thresholds or quotas, to minimize externalities and maximize gains for all parties in the long run (Madani & Dinar, 2011). This paper therefore introduces a threshold or extraction target via a collective choice mechanism into the shuffling scenario, following the work of Walker et al. (2000). The collective choice mechanism establishes a setting in which group members propose, communicate, and unanimously vote on the extraction target (norm) that a group will adopt. The target setting process unfolds in three steps. Firstly, at the start of each period, every group member proposes a target. Secondly, group members entered into a short interaction or communication. Finally, they vote on the proposals using a unanimity rule. 6

Social capital may influence the dynamics of the group target setting process. Consider a group of five participants from an indigenous community and their social capital at a group level. Since they are from the same indigenous community, this group of participants will have had interaction beforehand as compared to groups with members from different communities, which is often associated with bonding social capital. Evidence indicates that social capital plays a key role in fostering a stable level of cooperation in a setting where collective action problems, as illustrated in the shuffling scenario, are present (Bowles & Gintis, 2002; Ostrom & Ahn, 2009a). For example, a higher bonding dimension of social capital facilitates open and smooth communication among group members during the pre-voting communication stage. Simultaneously, communication can foster a sense of group identity and build trust (cognitive dimension of social capital) among group members (Shankar & Pavitt, 2002), which in turn reduces opportunistic behaviors like harvesting more units after convincing other members to set a lower target. Therefore, the first hypothesis of this study is that the higher the group’s cognitive and bonding social capital, the closer the group target per round is to the theoretical optimum for sustainable extraction under the no-shuffling scenario. Social capital improves a group’s ability to coordinate its actions in achieving socially optimal extraction levels that, in the absence of collective action, can only be realized under long-term property rights.

### Social Capital and Adherence to the Group Target

After the non-binding group target is established, group members undertake individual extraction decisions simultaneously and get private benefits according to the payoff function presented in equation (4.6). Group members undertake extraction under two scenarios: without revealing individuals’ extraction decisions (voting shuffling) and with revealing individuals’ extraction decisions (voting shuffling reputation). In the voting shuffling scenario, individuals privately and anonymously decide how many wounds to apply. That means, after each round, only the total number of wounds applied by a group is revealed to all group members, so information about group target violations is private and not shared. On the other hand, in the voting shuffling reputation scenario, individuals’ anonymity is removed, and individual extraction decisions are no longer private.

In the absence of a binding agreement or an enforcement mechanism (monitoring backed up by graduated sanctions), social capital can play a role in influencing individuals to abide by the group target. For example, under the voting shuffling scenario, cognitive social capital may create a social obligation (Ballet et al., 2007; Luo et al., 2018; Ostrom, 1990; Pretty, 2003; Pretty & Ward, 2001; Taylor, 1982). Concurrently, increased bonding social capital from increased interaction among group members enhances trust among group members and encourages members to observe their decisions and comply with the group target. Therefore, this paper hypothesizes that the higher the cognitive and bonding social capital, the closer individuals’ extracts are to the group target.

On the other hand, when anonymity is removed, as in the voting shuffling reputation scenario, the actions of each member are observable. Since group members who interact in the experiment come from the same community, they are likely to interact in the future. Through these repeated interactions, each member can get information about another member’s behavior. Consequently, a member who decides to harvest above the group targets may lose the reputation of being trustworthy. In response, group members may impose graduated sanctions on such members, such as social disapproval or group exclusion. By the same token, individuals who are trustworthy presumably have a better reputation and therefore have more to lose by not being cooperative. Moreover, bonding social capital facilitates individuals’ vulnerability to social sanctions within the group because they lose status if they violate the target. In such a context, even a very selfish member may not deviate from the target. Hence, this paper hypothesizes that cognitive and bonding social capital are more influential in a setting where reputation is a factor (voting shuffling reputation scenario) in decision-making processes compared to a setting where reputation is not considered (voting shuffling scenario).

## The Experiment and Survey Design

### The Experiment

The framed-field experiment was originally designed to test institutional arrangements for organizing the sustainable use of forest resources (Tegenie et al., 2024). The experiment was conducted in February 2021 in 11 kebeles (lower administration units in Ethiopia) of Kurmuk district, located in the Asosa zone of the Benishangul-Gumuz region.^7^ In these kebeles, forest management was handed over to the Berta community, and frankincense harvesting is successfully integrated with farming and traditional gold-mining activities, diversifying and sustaining dryland livelihoods. As a result, forests have great socioeconomic importance but face collective action problems on a regular basis because of the short-term property rights regime over shared forest resources.

Participants in the experiment were recruited from all 11 kebeles based on their membership in cooperatives and involvement in frankincense harvesting. Lists of members who engaged in frankincense harvesting were obtained from each kebele’s chairperson and used to randomly select and invite participants for the experiment. Within the cooperatives under the PFM program, the selected individuals were then randomly allocated into groups of five persons. Finally, the groups were randomly distributed into predetermined experimental sequences.

Each group of individuals participated in a CPR extraction game over six sessions of 3 rounds, totaling 18 rounds. Each session represented a harvesting cycle of the resource, which is designed to reflect the model of CPR extraction limited to three periods (i.e. rounds). The number of sessions and rounds was explained to the participants before the start of the experiment.

This study focuses only on the treatment conditions where participants propose and unanimously vote on the extraction targets with (voting shuffling reputation) and without revealing individual extraction decisions (voting shuffling), within a short-term property rights regime. This sub-sample of the full field experiment consists of 210 individuals that played the voting shuffling and voting shuffling reputation scenarios, allocated over 7 experimental sequences (Table 1).^8^ All sequences started with two shuffling sessions (i.e. short-term property rights scenario), but these data are not used for this chapter.

**Table 1:** Sequences of treatments

Session	Sequence						
	1	2	3	4	5	6	7
1	Shuffling	Shuffling	Shuffling	Shuffling	Shuffling	Shuffling	Shuffling
2	Shuffling	Shuffling	Shuffling	Shuffling	Shuffling	Shuffling	Shuffling
3	Voting shuffling	Voting shuffling reputation	Voting shuffling			Voting shuffling	Voting shuffling
4	Voting shuffling	Voting shuffling reputation	Voting shuffling			Voting shuffling	Voting shuffling
5	Voting shuffling	Voting shuffling	Voting shuffling Reputations	Voting shuffling	Voting shuffling		
6	Voting shuffling	Voting shuffling	Voting shuffling reputation	Voting shuffling	Voting shuffling		

The participants were all farmers who typically use the *Boswellia papyrifera* woodland for frankincense harvesting. The experimental task is framed as a decision on the number of wounds individuals want to apply to jointly owned *Boswellia papyrifera* trees to harvest frankincense. The participants are thus familiar with the context and the tasks demanded in the experiment, which enhances the external validity of the experiment. Cards are used to reflect the wounds to be applied to a tree, following Reichhuber et al. (2009), who used cards to represent beehives used for honey harvesting from jointly owned mountain forests in Ethiopia. The maximum number of cards is 40.^9^

The participants also played a pilot round to familiarize themselves with the task. At the start of the first round, two envelopes were given to each participant: one containing 40 cards and one empty envelope, representing “bags” where individuals keep their harvest. For every period representing the harvesting season, we asked participants to decide on the number of cuts they wanted to make (i.e., the number of cards they wanted to remove). Subjects are asked to remove the appropriate number of cards from the “tree envelope” and place them in the “harvest envelope.” Next, harvest envelopes were collected, and the decisions of all participants were recorded. Finally, an individual’s payoff was calculated using equation (4.6), and the parameters are set as follows: the initial health of a tree is 21, the production coefficient of wounding effort (ƶ) is equal to 0.7, the marginal effect of wounding a tree on its health (ɓ) is equal to 0.5 (the same as the cost of harvesting), and the price (P) is 5 tokens (Tegenie et al., 2024).

Participants made extraction decisions under two collective-choice scenarios of the shuffling scenario: voting shuffling and voting shuffling reputation.

#### Voting Shuffling Scenario

1. Property right duration: short-term. A group of five individuals jointly owns five trees. For extraction purposes, a tree envelope was randomly allocated to each individual for each round. To reflect the common good nature of trees, the tree envelopes containing decision cards (i.e., the cards remaining after playing a round) were collected from participants, shuffled, and randomly redistributed within a group. Participants kept the same harvest envelope throughout.
2. Collective choice: anonymous. Every group member proposes how much the members should extract. The proposals are limited to integers in the range of [0, 40]. Once proposals are made, they are listed anonymously on paper (identical proposals are listed only once). Group members were then allowed to communicate for five minutes and then choose one of the proposals through unanimous voting. Group members were informed that if an individual extracts more cards than the target, they will reduce the benefits for others. The aggregate number of cards removed was revealed to the group, but not the individual decisions. Each participant’s payoff was also communicated privately.

#### Voting Shuffling Reputation Scenario

1. Property right duration**:** short-term, similar to voting shuffling.
2. Collective choice: revealed. This scenario extended the voting shuffling scenario by publicly revealing everyone’s wound levels to the entire group. The group was aware of each participant’s extraction per period and who deviated from the established target. Group members were informed that if an individual extracts more than the established target, group members may gossip about it, which might affect the defector’s reputation within the group or community in general.

Under these two scenarios, outcome variables were collected at both the group and individual levels. At the group level, this study collected the group target per round, while at the individual level, the extraction levels per round were collected. All 210 participants completed the full experiment of six sessions, providing a panel data set with a total of 810 round-specific and 600 session-specific observations at the individual level and 162 round-specific and 120 session-specific observations at the group level.

### Survey Design

This study adopted questions from Krishna and Shrader (1999) and Grootaert and Bastelaer (2001) to measure the social capital of indigenous communities engaged in frankincense harvesting. Table 2 presents the questions asked to measure both structural bonding and cognitive social capital. For cognitive social capital, participants were asked about trust, reciprocity, and social norms. To measure trust, respondents were asked to rate the statement ‘*Generally speaking, most people in your village are honest and can be trusted*’ on a 4-point Likert scale. Reciprocity was measured with four questions that asked respondents about their willingness to help each other. Social norms were assessed with four questions that asked respondents’ willingness to abide by societal rules.

**Table 2:** Dimensions of social capital associated with 210 frankincense harvesters (mean values on a 1-4 scale)

Social capital dimensions	Description	Mean	S.D.
Cognitive			
Trust	Most people in your village are honest and can be trusted.	3.24	0.85
Reciprocity	Most people in your village are concerned for their welfare	2.36	0.96
	You get help from friends when needed (there is always someone in this village to help you).	3.37	0.87
	Most people in your village would try to take advantage of you if you were not alert.	2.67	0.89
	If you lose a goat, someone in your village will help you find it or return it to you if she or he finds it.	3.40	0.77
Social norms	You do pay attention to what others think or say about you.	2.54	0.97
	Most of the people in your village worry about their social status.	3.14	0.95
	You do anything your partner or villagers would say is wrong.	3.18	1.11
	You feel accepted as a member of your village.	3.52	0.59
Bonding			
Participation	In the past 12 months, how often did you join in village projects (e.g., tree planting)?	2.98	0.88
	In the past 12 months, how often did you take part in voluntary activities as a volunteer?	3.99	0.21
	Frequency of prayer per day with your family, neighbors, or other members of your village.	3.64	0.89
Membership	Average membership in an organization.	2.54	0.50

The study measures structural bonding social capital with four questions regarding participants’ involvement in local organizations and participation in local activities.^10^ The organizations were not limited to those directly related to forest management, considering the challenges of distinguishing relevant organizations due to the multi-objective nature of local community associations. Responses to both cognitive and bonding social capital are coded and given a score ranging from one to four.^11^

To standardize each indicator of cognitive (trust, reciprocity, and social norms) and bonding (membership and participation in local projects) social capital, the study transforms these indicators into normalized scores with a mean of 0 (where *z* < 0 reflects below average social capital) and a standard deviation (S.D.) of 1. The unweighted average scores are used as an index of individual cognitive and bonding social capital. Individual-level social capital indexes are then aggregated to measure group-level social capital. Additionally, the aggregate social capital of a group or individual is determined by summing up the group’s or individual’s cognitive and bonding social capital scores.

Additionally, the survey included participants’ economic and socio-demographic characteristics (Table 6 in the Appendix). The participants are homogenous in terms of socio-cultural characteristics: all are Muslims, 54% of the participants are female, the average age is 38, the average number of years of schooling is 2.8, and the average family size is 5.4. Their average annual household income was Birr 46,980 ($1,188), with Birr 8,478 ($210) coming from the frankincense business. This falls well below the poverty-line income level of $2.15 per day per person. For the majority of the participants, agriculture serves as the primary source of income, while a few reported traditional gold mining and non-timber forest product harvesting as the main sources. As per the local wealth classification, most participants (87%) characterize themselves as part of the middle class.

### Empirical Strategy

This study first tests the hypothesis that cognitive and bonding social capital influence group decisions to establish a target closer to the theoretical social optimum level. This is tested at the group level, with dependent variable ${N}_{j}$ representing the target set by group *j*, measured at either the round (*R*) and the session (*S*) level.

9 $${N}_{{jR}}=\,{\beta }_{0}+{\beta }_{1}T+{\beta }_{2}{{Sc}}_{j}+\mathop{\sum }\limits_{n=2}^{3}{\beta }_{3,n}{R}_{n}+\mathop{\sum }\limits_{n=2}^{3}{\beta }_{4,n}{R}_{n}* {{Sc}}_{j}+{{\beta }_{5}{Sc}}_{j}* T+{\beta }_{6}{X}_{j}+{\varepsilon }_{{jR}},$$

10 $${N}_{{jSn}}=\,{\beta }_{0}+{\beta }_{1}T+{\beta }_{2}{{Sc}}_{j}+\mathop{\sum }\limits_{n=4}^{6}{\beta }_{3,n}{S}_{n}+{{\beta }_{5}{Sc}}_{j}* T+{\beta }_{6}{X}_{j}+{\varepsilon }_{{jS}},$$

where, *Sc* refers to social capital, either cognitive or bonding. The round level regressions shed light on the decision making within sessions, and whether social capital influence the gradient in target setting and extraction across rounds. The parameters *β*_*2*_ and *β*_*4*_ capture the effects of social capital for each round and session (*β*_*2*_ for round 1 and *β*_*4*_ for rounds 2 and 3). The session level regression parameter *β*_*2*_ reflects the relationship between social capital and the level of extraction over the lifetime of the trees. In both models, *T* refers to the voting shuffling reputation scenario, with *β*_*1*_ capturing the impact of the voting shuffling reputation scenario on outcome variables, and *β*_*5*_ shows the influence of reputational concerns on the role of social capital for extraction decisions. The control variable *X* includes gender, age, years of schooling, social class, landholdings, income, and family size.

The second hypothesis, whether cognitive and bonding social capital influence individuals’ decisions to extract closer to the established group target, is tested at the individual level. Since the group targets vary across rounds and sessions, and are also influenced by social capital, observed deviations from the group targets can be due to both changes in group decisions or individual extraction levels. Hence, the specification considers the extraction level of individual *i* of group *j* at both round and session as the dependent variable (${Y}_{{ij}}$) and includes the group target (${N}_{j}$) in the model as an explanatory variable to show the correlation between the group target and extraction level:

11 $${Y}_{{ijR}}=\,{\beta }_{0}+{\beta }_{1}T+{\beta }_{2}{{Sc}}_{{ij}}+\mathop{\sum }\limits_{n=2}^{3}{\beta }_{3,n}{R}_{n}+\mathop{\sum }\limits_{n=2}^{3}{\beta }_{4,n}{R}_{n}* {{Sc}}_{{ij}}+{{\beta }_{5}{Sc}}_{{ij}}* T+{\beta }_{6}{X}_{{ij}}+{\beta }_{7}{N}_{{jR}}+{\varepsilon }_{{ijR}},$$

12 $${Y}_{{ijS}}=\,{\beta }_{0}+{\beta }_{1}T+{\beta }_{2}{{Sc}}_{{ij}}+\mathop{\sum }\limits_{n=4}^{6}{\beta }_{3,n}{S}_{n}+{{\beta }_{5}{Sc}}_{{ij}}* T+{\beta }_{6}{X}_{{ij}}+{\beta }_{7}{N}_{{jS}}+{\varepsilon }_{{ijS}},$$

where, ${\beta }_{7}$ captures individual adherence to the group extraction target. All models account for group random effects, to account for correlation of error terms rounds or sessions.

Endogeneity is a concern in both models due to response bias (i.e., respondents may provide socially desirable responses), unobserved heterogeneity, or reverse causality between the outcome variables and social capital. For example, an increased payoff from harvesting at the optimal level can strengthen cooperation among group members, leading to a higher level of social capital and vice versa. While social capital is not random, the reputation treatment is. It is therefore expected that any bias is similar for the different treatment groups; hence, the interaction term should identify the differential effects of reputational concerns on the association between social capital and extraction. Therefore, β_5_ in all equations can be interpreted as a casual effect. The other coefficients need to be interpreted as correlations.

## Results and Discussion

### Participants’ Characteristics and Social Capital

Before investigating the effect of social capital on target setting and extraction, it is useful to show the differences among participants in terms of their social capital (without specific hypotheses). Table 3 presents the association between individuals’ characteristics and their social capital. The results show that the cognitive dimension of social capital is higher among participants with a higher average annual income. Family size shows a statistically significant relationship with both cognitive and structural bonding dimensions of social capital, consistent with previous findings (Lee et al., 2017; Sheikh et al., 2015; Uphoff, 2000). This might be explained by the fact that larger families contribute more labor to community activities, such as collective farming, resource management, or other communal tasks, which in turn help build their social capital.

**Table 3:** Regression estimates for social capital dimensions (OLS)

Variables	Cognitive	Bonding
Age of participant	0.002	0.009
	(0.015)	(0.006)
Year of schooling	0.029	0.070^***^
	(0.051)	(0.019)
Household landholding	0.028	-0.070^***^
	(0.064)	(0.024)
Participants social class	0.469	-0.084
	(0.408)	(0.152)
Household income	0.117^**^	0.024
	(0.050)	(0.019)
Family size (number of adults + 0.5*children)	0.263^***^	0.116^***^
	(0.058)	(0.022)
Female (share)	0.392	1.789^***^
	(0.293)	(0.109)
Constant	-2.694^***^	-1.931^***^
	(0.795)	(0.296)
*R*^2^	0.048	0.276
Number of participants	210	210

Note: Statistical significance: ***1%,**5%, and *10%. Standard errors are in parentheses.

In addition, participants with higher levels of education exhibit higher levels of bonding dimension of social capital, which is also in line with previous studies (Larsen et al., 2004; Mathews, 2021). However, in contrast to Sheikh et al. (2015), participants with higher landholdings have a lower level of bonding dimension of social capital. This may be attributed to their busy schedules (i.e., farming) and limited time for participating in local organizations and activities. On the other hand, the bonding dimension of social capital is higher among female participants, consistent with previous studies (Lee et al., 2017; Paul et al., 2016).

### Group Target and Extraction Results

**Fig. 1:**
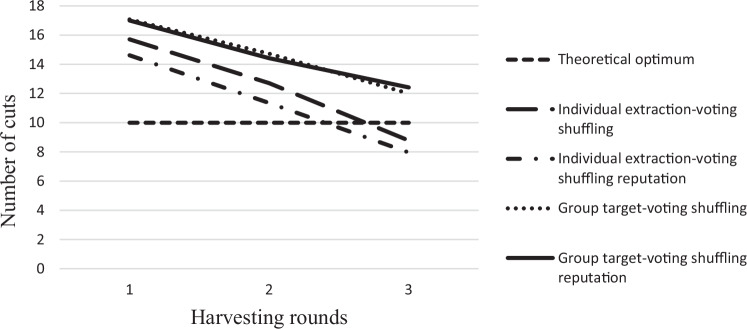
Average group targets and individual extraction per round for both the voting shuffling and voting shuffling reputation scenarios

Figure 1 presents the average group targets and extraction levels per round for both the voting shuffling and voting shuffling reputation scenarios. The horizontal dashed line indicates the theoretical optimal outcome under long-term property rights. For both scenarios, the average group targets show an almost identical pattern and are higher than the theoretical optimal level, in line with expectations under short-term property rights. Moreover, over time, group targets decline, due to initial over-extraction and the negative long-term externalities.

Similar to the group targets, individuals’ average extraction declines across periods as tree health deteriorates. The average extraction level across rounds under both the voting shuffling and voting shuffling reputation scenarios is lower than the group target. The extraction-target gap is wider for the voting shuffling reputation scenario, and the mean difference is statistically significant (*t*-value = 2.30). This suggests that reputation concern can be leveraged to reduce over-extraction to some extent, although it is not sufficient to meet the social optimum (see also Tegenie et al., 2024b).

### Social Capital, Collective Choice, and Individual Extraction

With regard to the group target setting, this study first tests the hypothesis that the higher the group’s cognitive and bonding dimensions of social capital, the closer the group’s target is to the theoretical optimum per round. The findings from the round-level analysis are presented in Table 4. Given that the average extraction targets are always above the theoretical optimum, a positive coefficient can be interpreted as moving away from optimum.^12^

**Table 4:** The association between group target setting and dimensions of social capital

	(1)	(2)	(3)	(4)	(5)
Cognitive dimension	0.624***		0.615***	-1.190	-1.095
	(0.166)		(0.169)	(1.409)	(1.354)
Cognitive*Period 2	-0.158		-0.168	-0.168	-0.168
	(0.171)		(0.169)	(0.170)	(0.174)
Cognitive*Period 3	-0.182		-0.194	-0.189	-0.189
	(0.245)		(0.237)	(0.238)	(0.244)
Bonding dimension		-0.160	-0.028	0.958	0.697
		(0.575)	(0.509)	(1.944)	(1.952)
Bonding*Period 2		-0.888*	-0.905**	-0.905**	-0.905*
		(0.460)	(0.449)	(0.452)	(0.462)
Bonding*Period 3		-1.117***	-1.137***	-1.134***	-1.131***
		(0.383)	(0.370)	(0.371)	(0.380)
Voting shuffling reputation			-0.026	-0.006	0.048
			(0.857)	(0.788)	(0.795)
Cognitive*Voting shuffling reputation				0.583	0.575
				(0.461)	(0.442)
Bonding*Voting shuffling reputation				-0.324	-0.251
				(0.581)	(0.585)
Period (ref =1)					
2	-2.427***	-2.430***	-2.431***	-2.431***	-2.431***
	(0.515)	(0.489)	(0.491)	(0.494)	(0.506)
3	-5.288***	-5.302***	-5.299***	-5.291***	-5.288***
	(0.646)	(0.620)	(0.623)	(0.628)	(0.643)
Constant	17.399***	17.410***	17.407***	17.405***	19.511**
	(0.489)	(0.560)	(0.485)	(0.488)	(4.666)
Control variables	No	No	No	No	Yes
R^2^(overall)	0.381	0.311	0.423	0.436	0.451
Observations	162	162	162	162	162

Note: Statistical significance: ***1%,**5%, and *10%. Robust standard errors are in parentheses (clustered at group level). All models account for group random effects. Controls variables include: gender, age, years of schooling, social class, landholdings, income, and family size.

Columns 1 and 3 show that the coefficients for the cognitive dimension of social capital are positive and statistically significant. This effect is constant across rounds, with a statistically significant coefficient in period 1 that stabilizes in the subsequent rounds. This suggests that, in contrast to the hypothesis, the cognitive dimension of social capital is associated with a wider gap between observed group targets and the optimum. During the experiment, information on the socially optimal level of 10 units was not provided to the group members. One explanation could be that, as groups’ cognitive dimension of social capital increases, groups may decide to set a higher targets to avoid imposing strict limitations that could lead to dissatisfaction or conflict among members who might feel constrained by lower targets. Supporting these findings, a study conducted in the Southwestern Highlands of Uganda showed that social capital enhances the capacity to resolve conflicts through mediation and negotiation within groups (Sanginga et al., 2007). Other evidence also indicates that social capital influences the socialization of group members, promoting conflict avoidance and empathy (Hornsey et al., 2006) (Tables 7,8).

The session-level regressions confirm that the round-level estimates for cognitive social capital also aggregate to higher targets over the full session (the session-level results are presented in the appendix Table 9). Similarly, Aida (2019) shows that in Sri Lankan irrigation systems, strong social ties can reinforce locally accepted extraction practices, which may not always align with sustainable use.

In contrast, bonding social capital is associated with a reduced gap between actual and optimal target at session level, in line with the hypothesis. But the effect of the structural bonding dimension of social capital only materialized in period 2 and then increased slightly in period 3. This may be because, as resources become scarcer, the bonding dimension of social capital facilitates coordination among group members, thereby leading to a reduction in the group target towards the optimum. This aligns with previous studies indicating that collective actions are more prevalent in resource-scarce conditions (Aida, 2019; Hayami, 2009; Ramirez-Sanchez & Pinkerton, 2009).

However, as more variables are included in the random effect regression analysis (including the interaction term), the association between the target and cognitive dimension of social capital disappears (see columns 4 and 5 of Table 4) — including more variables may capture additional variation in the dependent variable.

The coefficients for the interaction of the voting shuffling reputation scenario with both cognitive and bonding dimensions of social capital are not statistically significant (both at round- and session-level analysis). This suggests that whether individual extraction decisions are publicly revealed or kept hidden, the effects of both the cognitive and bonding dimensions of social capital on the target remain the same (see Appendix, Tables 10 and 11 for the aggregated effect of social capital) (Table 12).

Table 5 presents the association between round-specific extraction and the different dimensions of social capital. The regression results show that there is only partial adherence to group decisions as changes in group targets are adopted for 20 percent in individual decisions. Regarding extraction, the second hypothesis posits that a higher level of an individual’s cognitive and bonding dimensions of social capital would lead to individuals’ extraction being closer to the group target. However, the session-level analysis results show that both the cognitive and structural bonding dimensions of social capital are not associated with individual extraction levels (see Appendix, Table 13), which is not in line with the hypothesis.

**Table 5:** The association between individual extraction decisions and social capital

	(1)	(2)	(3)	(4)	(5)
Group target	0.199***	0.199***	0.199***	0.200***	0.202***
	(0.028)	(0.029)	(0.027)	(0.027)	(0.024)
Cognitive dimension	0.104**		0.103**	0.289*	0.313
	(0.045)		(0.045)	(0.161)	(0.191)
Cognitive*Period 2	-0.195***		-0.195***	-0.195***	-0.195***
	(0.052)		(0.053)	(0.053)	(0.052)
Cognitive*Period 3	-0.177***		-0.178***	-0.178***	-0.178***
	(0.067)		(0.067)	(0.067)	(0.052)
Bonding dimension		0.045	0.038	0.061	0.008
		(0.111)	(0.111)	(0.355)	(0.401)
Bonding*Period 2		-0.019	-0.009	-0.009	-0.008
		(0.144)	(0.141)	(0.141)	(0.123)
Bonding*Period 3		-0.009	-0.000	0.000	0.001
		(0.173)	(0.174)	(0.175)	(0.123)
Voting shuffling reputation			-0.920***	-0.922***	-0.945***
			(0.158)	(0.157)	(0.198)
Cognitive* Voting shuffling reputation				-0.059	-0.065
				(0.050)	(0.059)
Bonding* Voting shuffling reputation				-0.006	0.018
				(0.108)	(0.121)
Period (ref = 1)					
2	-2.961***	-2.960***	-2.962***	-2.959***	-2.954***
	(0.210)	(0.214)	(0.210)	(0.211)	(0.209)
3	-6.140***	-6.139***	-6.143***	-6.136***	-6.125***
	(0.297)	(0.301)	(0.291)	(0.293)	(0.238)
Constant	12.142***	12.144***	12.358***	12.334***	12.869***
	(0.505)	(0.520)	(0.489)	(0.493)	(0.676)
Control variables	No	No	No	No	Yes
R^2^	0.628	0620	0.638	0.638	0.642
Observations	810	810	810	810	810

Note: Statistical significance: ***1%,**5%, and *10%. Standard errors are in parentheses (clustered at a group level). All models account for group random effects. Controls variables include: gender, age, years of schooling, social class, landholdings, income, and family size.

Nevertheless, the round-level analysis results in Table 5 show that the coefficients for the cognitive dimension of social capital are statistically significant. The gradient becomes steeper, suggesting that the cognitive dimension of social capital moves individuals to a more short-term focus in their extraction decisions. The structural bonding dimension of social capital does not show a statistically significant association with extraction in any period. One possible explanation for the deviation of the observed coefficients from the expected behavior is methodological: aggregation at the session level may obscure behavioral variation occurring across rounds. Averaging behavior across sessions may reduce variability and statistical power (Alker Jr, 1969; Clark & Avery, 1976). Micro-level analysis offers a potential solution, as round-level analysis captures short-term behavioral adjustments, making the influence of cognitive social capital on extraction decisions detectable (Tables 14–16).

The third hypothesis is that the cognitive and bonding dimensions of social capital are more influential in the voting shuffling reputation scenario compared to the voting shuffling scenario. The coefficient for the voting shuffling reputation scenario is negative and statistically significant, replicating previous findings (Tegenie et al., 2024). Nonetheless, the coefficients for the interaction of the voting shuffling reputation scenario with both cognitive and bonding social capital (at both the round- and session level) are not statistically significant, implying that whether individual information is disclosed or not, the effects of both cognitive and structural bonding dimensions remain the same. This may be explained by the already dominant direct effect of information disclosure and reputation concerns on resource extraction decisions, overriding any influence of cognitive and bonding social capital in modifying behavior in response to the disclosed information. Another possibility is that the interaction effects are too weak to be detected.

I expected that higher social capital—particularly its cognitive component—would promote the sustainable use of natural resources, leading both group targets and individual extractions toward more sustainable levels. However, contrary to expectations, I found that cognitive social capital is associated with higher extraction targets, yet individuals do not adhere to these targets and extract less than intended. This suggests that cognitive social capital is not linked to increased extraction, ruling out the potential negative effects of social capital, such as strong in-group trust combined with weak external accountability, that can lead to over-extraction (Ostrom,^1990^). One possible explanation for the rise in targets is that increased cognitive social capital may make individuals reluctant to confront or penalize their group members, as they do not want to jeopardize relationships by enforcing targets. This may be especially salient if the details of the payoff function have not been revealed, and everyone is simply making their best (informed) guess. In that case, high social capital may lead them to be more empathetic toward group members’ suggestions, even if they differ from their own. As a result, they may be less motivated to insist on lowering the targets. If this interpretation holds, then providing information about the payoff function to the group (or, in real life, educating them on sustainable extraction practices and what they mean for their long-term income) might still leverage cognitive social capital to improve sustainable extraction.

## Conclusions

This study explores how the cognitive and bonding dimensions of social capital influence the target-setting process and extraction decisions of communities that hold short-term property rights over CPR. It evaluates target-setting behavior by comparing it to the social optimum level and the extraction decision relative to the established target. From the findings of this study, it can be concluded that the cognitive dimension of social capital facilitates cooperation but not sustainable use of resources, as it steers group decisions away from the optimum level and influences individuals to concentrate more on immediate gains under short-term property rights regime. However, the influence of structural bonding social capital is contingent upon the circumstances the group is facing, such as resource scarcity. Finally, information disclosure does not alter the influence of both the cognitive and structural bonding dimensions of social capital.

These results are consistent with evidence from other contexts—for example, Sri Lanka (Aida, 2019), China (Baylis et al., 2018), and India (Knuffman, 2011)—and underscore the broader role of social capital in shaping resource-use behavior, though its influence may vary depending on the ecological and institutional context.

The findings of this study have policy implications. The government of Ethiopia is currently scaling up community-based forest resource management, predominantly in the drylands of the country (Amenu, 2019; Beyene, 2015; Walle & Nayak, 2022; Zerga et al., 2019). Property rights over dryland forest resources are particularly complex, especially the use rights for high-value resources like frankincense-bearing trees, which are insecure, largely unregulated, and expensive to regulate. In light of these challenges, strengthening community ties through targeted interventions and implementing complementary institutional measures tailored to the specific needs of PFM project areas can serve as an alternative mechanism to foster cooperation.

The study is subject to some limitations. First, the study acknowledges that social capital may influence participants’ decisions differently if information on the socially optimal level of extraction is provided, and this may be a topic for future research. Secondly, with the increase in cognitive social capital, groups set higher targets, while individual members continue to harvest below these targets. The reasons behind this behavioral pattern require further investigation. Third, the study acknowledges that collective social capital may not be solely determined by aggregating individuals’ social capital since the sources and benefits of social capital available at the individual level may differ from those at the group level. Fourth, other dimensions of structural social capital, such as bridging and linking, may influence group or individual decisions in collective action. Future research, using multiple indicators, can identify how forest resource use is linked to bridging and linking dimensions of social capital. Finally, the social capital variable is unlikely to be exogenous, which may introduce endogeneity bias. Future studies may focus on methods for drawing a causal inference between social capital and the outcome variables in the same context.

## Data Availability

Data will be provided on request.

## Appendix: Additional tables

Tables 6–16

**Table 6:** Descriptive statistics of participants’ characteristics

	Mean	Standard deviations	Minimum	Maximum
Participant characteristics				
Age (years)	38.46	11.52	18.00	68.00
Years of schooling	2.80	3.28	0.00	12.00
Length of residency in the village (years)	33.59	13.13	7.00	65.00
Landholding (ha)	2.75	2.33	0.00	10.00
Number of adults over 18 years	3.10	3.09	0.00	8.00
Number of children under 18 years	4.63	3.29	0.00	17.00
Family size (number of adults + 0.5*children)	5.42	2.72	1.00	13.50
Gender (female) (%)	54.00			
Participant’s main economic activities (%)				
Farming	75.20			
Livestock	4.76			
Traditional gold mining	14.8			
Non-timber forest product harvesting (Frankincense, honey)	4.76			
Annual income (ETB)	46980.32	141256.60	2200	2010000.00
Income from frankincense (ETB)	8478.40	12039.93	0.00	149000.00
Participation in off-farm activities (%)				
Yes	19.56			
Social class (%)				
Poor	13.33			
Middle class and above	86.67			
Age of PFM (years)	6.14	2.04	2.00	9.00

Note**:** Standard deviations are presented in parentheses

**Table 7:** Mean comparison results (round-specific)

Scenario	Target		Extraction level	
	Mean	S.D.	Mean	S.D.
Voting shuffling	14.83	4.09	12.27	3.91
Voting shuffling reputation	14.61	4.64	11.31	3.50
Combined	14.78	4.21	12.06	3.84
Mean difference	0.22		0.96	

Standard deviations are presented in parentheses

**Table 8:** Mean comparison results (session-specific)

Scenario	Target		Extraction level	
	Mean	S.D.	Mean	S.D.
Voting shuffling	43.80	9.48	36.78	4.78
Voting shuffling reputation	43.83	11.46	33.93	4.34
Combined	43.81	9.88	36.21	4.83
Mean difference	0.03		2.85

**Table 9:** The association between group-target and dimensions of social capital (session-specific)

	(1)	(2)	(3)	(4)	(5)
Cognitive dimension	1.589***		1.566***	-2.392	-2.746
	(0.267)		(0.259)	(3.873)	(3.744)
Bonding dimension		-2.919***	-2.910***	0.859	1.078
		(1.062)	(1.045)	(7.159)	(7.300)
Voting shuffling reputation			-0.398	-0.388	-0.373
			(2.080)	(2.068)	(2.155)
Cognitive* Voting shuffling reputation				1.268	1.386
				(1.264)	(1.205)
Bonding*Voting shuffling reputation				-1.182	-1.248
				(2.064)	(2.160)
Session (ref =3)					
4	-0.533	-0.533	-0.777	-0.650	-0.561
	(2.516)	(2.687)	(2.568)	(2.448)	(2.504)
5	1.023	1.266	1.552	1.505	1.465
	(2.171)	(2.425)	(2.183)	(2.164)	(2.206)
6	0.606	0.818	0.964	1.012	1.002
	(2.290)	(2.391)	(2.131)	(2.171)	(2.278)
Constant	43.891***	43.484***	43.782***	43.730***	44.123***
	(1.733)	(1.875)	(1.785)	(1.732)	(12.651)
Control variables	No	No	No	No	Yes
R^2^	0.195	0.072	0.256	0.274	0.294
Observations	120	120	120	120	120

Note: Statistical significance: ***1%,**5%, and *10%. Standard errors in parentheses. All models account for group random effects. Controls variables include: gender, age, years of schooling, social class, landholdings, income, and family size.

**Table 10:** The association between group-target and aggregate social capital (round-specific)

Variables	(1)	(2)	(3)
Social capital (aggregate)	0.588^***^	-0.347	-0.545
	(0.165)	(1.130)	(1.061)
Social capital*period 2	-0.233	-0.233	-0.233
	(0.158)	(0.159)	(0.162)
Social capital*period 3	-0.282	-0.279	-0.279
	(0.222)	(0.224)	(0.229)
Voting shuffling reputation		0.020	0.021
		(0.842)	(0.853)
Social capital*Voting shuffling reputation		0.298	0.380
		(0.367)	(0.354)
Period (ref = 1)			
2	-2.428^***^	-2.428^***^	-2.428^***^
	(0.504)	(0.507)	(0.519)
3	-5.290^***^	-5.287^***^	-5.281^***^
	(0.636)	(0.640)	(0.656)
Constant	17.404^***^	17.404^***^	19.536^**^
	(0.496)	(0.487)	(4.656)
Controls	No	No	Yes
R^2^ (overall)	0.342	0.337	0.381
Round-specific observations	162	162	162

Note: Statistical significance: ***1%,**5%, and *10%. Robust standard errors in parentheses. All models account for group random effects.

**Table 11:** The association between group-target and aggregate social capital (session-specific)

Variables	(1)	(2)	(3)
Voting shuffling reputation	-0.162	-0.155	-0.424
	(2.354)	(2.428)	(2.518)
Aggregate social capital	1.182^***^	0.955	-1.328
	(0.276)	(3.413)	(3.329)
Social capital*Voting shuffling reputation		0.071	0.822
		(1.098)	(1.074)
Session (ref = 3)			
4	-0.533	-0.533	-0.533
	(2.638)	(2.646)	(2.619)
5	0.755	0.751	1.104
	(2.267)	(2.289)	(2.282)
6	0.340	0.337	0.728
	(2.422)	(2.436)	(2.330)
Constant	43.939^***^	43.938^***^	44.943^***^
	(1.738)	(1.741)	(11.187)
Controls	No	No	Yes
R^2^ (overall)	0.118	0.118	0.196
Observations	120	120	120

Note: Statistical significance: ***1%,**5%, and *10%. Robust standard errors in parentheses. All models account for group random effects.

**Table 12:** The association between extraction and social capital dimensions (session-specific)

	(1)	(2)	(3)	(4)	(5)
Group target	0.078	0.076*	0.086**	0.086**	0.088**
	(0.047)	(0.045)	(0.043)	(0.042)	(0.039)
Cognitive dimension	-0.018		-0.015	0.192	0.385
	(0.045)		(0.046)	(0.470)	(0.479)
Bonding dimension		0.010	0.013	0.049	0.111
		(0.104)	(0.099)	(0.898)	(0.973)
Voting shuffling reputation			-2.396**	-2.404**	-2.625***
			(0.947)	(0.935)	(0.823)
Cognitive*Voting shuffling reputation				-0.065	-0.120
				(0.151)	(0.154)
Bonding*Voting shuffling reputation				-0.010	-0.028
				(0.276)	(0.301)
Session (ref =3)					
4	-0.144	-0.158	-0.176	-0.205	-0.330
	(0.797)	(0.793)	(0.729)	(0.706)	(0.649)
5	-0.559	-0.561	-0.638	-0.629	-0.712
	(1.037)	(1.033)	(0.984)	(0.963)	(0.856)
6	-1.039	-1.046	-1.357*	-1.391*	-1.635**
	(0.901)	(0.895)	(0.777)	(0.768)	(0.775)
Constant	33.211***	33.306***	33.439***	33.441***	34.626***
	(2.035)	(1.949)	(1.946)	(1.911)	(1.950)
Control variables	No	No	No	No	Yes
R^2^	0.052	0.053	0.110	0.112	0.142
Observations	600	600	600	600	600

Note: Statistical significance: ***1%,**5%, and *10%. Robust standard errors in parentheses. All models account for group random effects.

**Table 13:** The association between extraction and aggregate social capital (round-specific)

Variables	(1)	(2)	(3)
Group target	0.194***	0.192***	0.193***
	(0.028)	(0.025)	(0.024)
Social capital	0.095**	0.225*	0.232
	(0.039)	(0.125)	(0.164)
Social capital*period 2	-0.166***	-0.166***	-0.166***
	(0.046)	(0.046)	(0.048)
Social capital*period 3	-0.150**	-0.151**	-0.151***
	(0.063)	(0.064)	(0.048)
Voting shuffling reputation		-0.924***	-0.943***
		(0.156)	(0.198)
Social capital*Voting shuffling reputation		-0.041	-0.041
		(0.038)	(0.050)
Period (ref = 1)			
2	-2.974***	-2.980***	-2.977***
	(0.210)	(0.210)	(0.208)
3	-6.167***	-6.182***	-6.176***
	(0.296)	(0.292)	(0.236)
Constant	12.230***	12.481***	12.923***
	(0.504)	(0.477)	(0.676)
Controls	No	No	Yes
R^2^ (overall)	0.626	0.636	0.640
Round-specific observations	810	810	810
Random effect model	Yes	Yes	Yes

Note: Statistical significance: ***1%,**5%, and *10. Standard errors in parentheses. All models account for group random effects.

**Table 14:** The association between extraction and aggregate social capital (session-specific)

Variables	(1)	(2)	(3)
Group target	0.077	0.085**	0.086**
	(0.047)	(0.042)	(0.039)
Social capital	-0.012	0.153	0.301
	(0.040)	(0.346)	(0.348)
Voting shuffling reputation		-2.408***	-2.592***
		(0.928)	(0.844)
Social capital* Voting shuffling reputation		-0.051	-0.094
		(0.109)	(0.110)
Session (ref = 3)			
4	-0.151	-0.207	-0.295
	(0.795)	(0.703)	(0.662)
5	-0.557	-0.626	-0.715
	(1.034)	(0.963)	(0.887)
6	-1.041	-1.383*	-1.570**
	(0.900)	(0.769)	(0.776)
Constant	33.260***	33.520***	34.640***
	(2.018)	(1.899)	(1.976)
Controls	No	No	Yes
R^2^ (overall)	0.052	0.112	0.140
Observations	600	600	600

Note: Statistical significance: ***1%,**5%, and *10%. Standard errors in parentheses. All models account for group random effects.

**Table 15:** The association between individual payoff and dimensions of social capital (round-specific)

Variables	(1)	(2)	(3)
Cognitive social capital	2.574*		-1.636
	(1.412)		(3.955)
Cognitive *period 2	-5.901***		-5.864***
	(1.863)		(1.878)
Cognitive*period 3	-4.006**		-3.950**
	(1.903)		(1.920)
Bonding social capital		1.271	6.487
		(2.662)	(5.725)
Bonding*period 2		-3.533	-3.162
		(4.695)	(4.671)
Bonding*period 4		-3.555	-3.313
		(3.776)	(3.788)
Voting shuffling reputation			12.186***
			(2.702)
Cognitive* Voting shuffling reputation			1.333
			(0.947)
Bonding*Voting shuffling reputation			-1.684
			(1.458)
Period (ref = 1)			
2	-407.195***	-407.351***	-407.216***
	(7.406)	(7.493)	(7.418)
3	-621.638***	-621.747***	-621.653***
	(6.888)	(6.951)	(6.911)
Constant	692.617***	692.683***	689.902***
	(4.753)	(4.801)	(5.040)
R^2^ (overall)	0.942	0.941	0.943
Observations	804	804	804

Note: Statistical significance: ***1%,**5%, and *10%. Standard errors in parentheses. All models account for group random effects.

**Table 16:** The association between individual payoff and dimensions of social capital (session-specific)

Variables	(1)	(2)	(3)
Session (ref =3)			
4	20.519	20.882	21.084
	(28.204)	(28.563)	(28.040)
5	23.809	24.670	26.259
	(29.338)	(29.456)	(28.177)
6	-18.265	-17.477	-13.135
	(30.769)	(31.117)	(26.835)
Cognitive dimension	1.760		-8.294
	(2.223)		(10.808)
Bonding dimension		-5.704**	-3.250
		(2.470)	(15.178)
Voting shuffling reputation			64.051***
			(13.913)
Cognitive* Voting shuffling reputation			3.175
			(3.033)
Bonding* Voting shuffling reputation			-0.788
			(4.265)
Constant	1029.378***	1028.570***	1014.563***
	(18.892)	(19.215)	(18.332)
R^2^	0.01	0.01	0.03
Observations	600	600	600

Note: Statistical significance: ***1%,**5%, and *10%. Standard errors in parentheses. All models account for group random effects.

## Footnotes

1 The Ethiopian Forest Development, Conservation, and Utilization Proclamation No. 1065/2018 defines PFM as ‘a forest management approach executed through an agreement between the state and the local community that inhabits inside or around the forest area over the management, protection, and utilization of forests owned by the state based on predefined responsibilities and benefit sharing mechanisms*.*'

2 Frankincense is an aromatic resin produced by slightly shaving the external layer of the bark of a tree and leaving the original tree intact.

3 For full details on the larger experiment, see Tegenie et al. (2024b).

4 The dimensions of social capital are various; other scholars have categorized them as structural, rational, and cognitive social capital (see Gooderham, 2007; Nahapiet and Ghoshal, 1998).

5 In an informal survey, frankincense harvesters mentioned that they spent a maximum of one hour opening (reopening) a wound on trees and collecting frankincense. During the harvesting season, they reopen a single wound a total of 12 times, which amounts to half a day (0.5 in the cost function).

6 In real-life situations, farmers make their own decisions where they can communicate with each other. Communication allows farmers to reach an agreement and vote the same way (unanimous voting). Scholars (e.g., Ostrom et al. (1994)) identify communication among individuals as a key factor in reaching agreements and developing rules for the use of the resource.

7 There are 16 kebeles in the Kurmuk district. The pilot experiment was conducted in two kebeles, and the actual experiment was conducted in nine kebeles.

8 A total of 330 individuals participated in the larger experiment, a setup that included both no shuffling and shuffling scenarios, either separately or in combinations. Monetary incentives, both individual and group-based, were also introduced into the shuffling scenario (the empty cell in Table 1). The sample size was determined based on power calculations. To control for kinship, no family members are placed in the same group. For full details see Tegenie et al. (2024b).

9 Practically, individuals apply on average 13.4 wounds per year to a tree with a diameter at breast height >=20 cm (Tegenie et al., 2024a). Given the parameters of the payoff function, the number of cards (40) does not impose any constraints.

10 All participants in the experiment engaged in their village projects, but there are variations in the level of their commitment and participation. Therefore, the level of participation is used as a proxy measure of bonding social capital. The frequency of voluntary activities is not included in the analysis since there is no variation among participants. Moreover, the study does not incorporate the bridging and linking dimensions of social capital due to a lack of reliable data.

11 For cognitive social capital, the scale ranges from 1 (strongly disagree) to 4 (strongly agree). For structural bonding, social activities are rated on a scale of 1 (never) to 4 (frequently), and membership is rated on a scale of 1 (no) to 4 (more than two). The study takes into account the negative connotation presented in Table 2 during data analysis, such that higher scores reflect higher levels of social capital for all indicators.

12 This is also reflected by the constants for all specifications, which are above the theoretical optimum of 10 in the round-level regressions and above 30 for the session-level regressions.
